# Effect of Edema Disease Vaccination on Mortality and Growth Parameters in Nursery Pigs in a Shiga Toxin 2e Positive Commercial Farm

**DOI:** 10.3390/vaccines9060567

**Published:** 2021-05-31

**Authors:** Susana Mesonero-Escuredo, Joaquín Morales, Raúl Carlos Mainar-Jaime, Gonzalo Díaz, José Luís Arnal, Carlos Casanovas, Sergio Barrabés, Joaquim Segalés

**Affiliations:** 1 CEVA Salud Animal, 08028 Barcelona, Spain; susana.mesonero-escuredo@ceva.com (S.M.-E.); carlos.casanovas@ceva.com (C.C.); sergiosopeira@gmail.com (S.B.); 2 PigCHAMP Pro Europa SL, 40006 Segovia, Spain; joaquin.morales@pigchamp-pro.com (J.M.); gonzalo.diaz@pigchamp-pro.com (G.D.); 3 Departamento de Patología Animal, Facultad de Veterinaria, Instituto Agroalimentario de Aragón-IA2-(Universidad de Zaragoza-CITA), 50013 Zaragoza, Spain; rcmainar@unizar.es; 4 Exopol, San Mateo de Gállego, 50840 Zaragoza, Spain; jlarnal@exopol.com; 5 Departament de Sanitat i Anatomia Animals, Universitat Autònoma de Barcelona (UAB), 08193 Barcelona, Spain; 6 Centre de Recerca en Sanitat Animal (CReSA, IRTA-UAB), Campus de la Universitat Autònoma de Barcelona, 08193 Barcelona, Spain; 7 OIE Collaborating Centre for the Research and Control of Emerging and Re-emerging Swine Diseases in Europe (IRTA-CReSA), 08193 Barcelona, Spain

**Keywords:** edema disease, vaccination, antibiotic reduction, productive parameters

## Abstract

Diseases caused by *Escherichia coli* are recognized as major problems in the swine industry, one of them being edema disease (ED). Importantly, the current decrease in antibiotic use may cause difficulties in controlling the disorders caused by *E. coli*. Therefore, this study assessed the efficacy of a commercial vaccine against ED in nursery pigs from a farm with previous history of ED. A total of 1344 pigs were monitored; half of them were randomly assigned to a vaccinated group (VG) and the other half to a non-vaccinated group (NVG). The vaccine was administered at 7 days of age. Animals received a pre-starter feed with 2500 ppm of zinc oxide (ZnO) for 2 weeks and a starter feed without ZnO for another 3 weeks. Pen-group weights were recorded at 28 (weaning), 42 (end of pre-starter phase), and 63 days of life (end of nursery phase). Death/culling rates, average daily gain (ADG), and average daily feed intake (ADFI) were calculated for each group at each phase. The overall relative risk of dying/being culled for a pig in the NVG was 5 times higher than that of the VG group but increased to 12 times higher during the starter period. ADG and ADFI were also significantly higher in the VG group for that period. Vaccination against ED significantly reduced pig losses and improved ADG and ADFI, particularly when ZnO was not used.

## 1. Introduction

Antibiotics have been generally used in swine production to reduce colonization of pathogenic bacteria in the gut. However, their contribution to the potential development of antibiotic-resistant strains of bacteria [1] prompted the European Union (EU) to implement a full ban on the use of antibiotics as growth promoters in livestock in January 2006 (Regulation 1831/2003/EC on additives for use in animal nutrition). This measure was first applied in Sweden and Denmark, leading to an important increase in the prevalence of postweaning diarrhea and mortality rates due to *Escherichia coli* infections [2]. The proliferation of this bacterium can cause a wide range of diseases in pigs, including postweaning diarrhea (PWD) and edema disease (ED). Postweaning diarrhea is usually associated with proliferation of enterotoxigenic *E. coli* (ETEC) [3]. This pathotype is characterized by the production of enterotoxins and adhesins, both of which essential for disease development [4]. Edema disease is caused by Shiga-toxin-producing *E. coli* (STEC), also known as edema disease *E. coli* (EDEC). These strains produce the Shiga toxin 2e (Stx2e) and may possess the fimbriae variants F18ab or F18ac [5].

Antibiotic treatment is still the most widely used therapeutic approach to improve the health condition of pigs affected with PWD and ED, colistin being one of the main antimicrobials used before the description of *mcr*-1 gene, the first reported plasmid gene encoding resistance to colistin [6]. Additionally, some feed management strategies, such as restriction of feed intake, reduction of crude protein and digestible energy, high fiber diets, and ad libitum feeding of fiber, have also been reported as effective in controlling *E. coli* infection outbreaks [7]. However, these measures have obvious detrimental effects on the performance of the animals. Another alternative for controlling *E. coli* infections has been the use of in-feed zinc oxide (ZnO), but the European Commission has finally decided to ban the therapeutic use of ZnO in feed by 2022 due to its contribution to the potential increase of antimicrobial resistance and environmental issues [8,9]. 

The Food and Agriculture Organization (FAO) has also emphasized the need to prevent infectious diseases in animals through several measures compiled into three main categories: good animal husbandry, effective biosecurity, and vaccination. The latter induces immune responses that confer protection against the target disease, but some evidence also suggests that immunization through vaccination may perturb the immune system in such a way that it might also increase protection against unrelated pathogens [10], the so called “non-specific effects” (NSE) and, in a wider concept, trained immunity [11]. To date, there are different registered vaccines in Europe for fighting against PWD and ED. There is a vaccine for reducing mortality and clinical signs due to PWD and early ED that includes different *E. coli* bacterins for the passive immunization of piglets [5]. Additionally, a bivalent live vaccine for controlling PWD that comprises F4 and F18 non-pathogenic *E. coli* strains does exist [5]. Finally, prevention of EDEC is feasible through licensed vaccines containing a genetically modified recombinant Stx2e [12]. 

Considering the permanent need to prevent and control *E. coli* infections in postweaning pigs and the need to minimize antibiotic usage, the objective of this study was to assess the global effects (on health and performance) of the use of a licensed vaccine against ED in a population of nursery piglets from a commercial farm with previous history of ED.

## 2. Materials and Methods

### 2.1. Farm Description

The study took place in a commercial 500-sow, farrow-to-nursery herd located in Segovia (Spain). The farm produced in a 3-week batch farrowing system, following all-in-all-out procedures. Piglets were weaned at 4 weeks of age and housed in the nursery barn for 5 weeks. The nursery barn included 7 identical rooms containing 24 pens (8 piglets/pen) each. Environmental parameters in the nursery barn were set up according to piglet needs. 

This farm had clinical history of ED during the starter phase (43–63 days), which was diagnosed based on clinical signs (nervous signs and sudden death) and laboratory analyses. STEC infection was identified by detection of genes coding for virulence factors Stx2e and fimbriae F18 by qPCR (EXOone *E. coli* virulence factors, Exopol, Spain) from jejunum content samples from previous ED outbreaks. 

When the study was conducted, the farm was positive but stable to porcine reproductive and respiratory syndrome virus (PRRSV) infection, positive to *Mycoplasma hyopneumoniae*, and positive to Porcine circovirus 2 (PCV2) but without evidence of clinical expression.

Estimated data on the performance of 480 piglets were recorded in the 8 months before the beginning of this study. These piglets received a commercial feed that included ZnO (2500 ppm) for the first 14 days and colistin (150 ppm) for 7 days (Table 1).

### 2.2. Study Design

A total of 1344 nursery pigs from 14 farrowing batches were considered for this study. At 7 days of age, 8 piglets (4 males and 4 females) from each litter were selected and ear-tagged after discarding the heaviest and the lightest ones by visual inspection by the farm veterinarian. Half of the litters were then randomly assigned to a vaccine group (VG) or a non-vaccine group (NVG) after matching by sow parity. Animals from the VG were vaccinated against ED (Ecoporc SHIGA^®^, CEVA, Barcelona, Spain) at that time, while animals from the NVG were left without vaccine. The dosage applied to piglets was a single intramuscular injection (1 mL) of a genetically modified recombinant Stx2e antigen for the active immunization of piglets from the age of 4 days onwards [13]. The onset of the immunity is 21 days after vaccination, and the duration of immunity 105 days after vaccination [13].

At weaning, animals were distributed in 168 nursery pens (8 piglets/pen), half with the VG piglets and the other half with those from the NVG. A commercial pre-starter feed that included ZnO (2500 ppm) was provided for the first 14 days postweaning and then a starter feed without ZnO for the last 3 weeks of the nursery period. All the animals were monitored along the whole nursery period (5 weeks). Since the study was carried out in a commercial farm, groups could not be blinded, but farm personnel were properly trained to guarantee that the same monitoring was performed for both groups during the whole nursery period. 

### 2.3. Data Collection

To measure growth performance parameters, i.e., average daily gain (ADG) and average daily feed intake (ADFI), piglets were weighed on a pen basis at 28 (weaning), 42 (end of pre-starter feeding), and 63 (end nursery phase) days of life. Feed intake in each pen was also measured in the same productive phases (days 28–42 and 43–63). 

Both culled and dead piglets were recorded throughout the nursery period. Date of death/culling, weight, and possible cause of death/culling were also registered. Data of dead/culled piglets were considered for growth performance parameter estimates within the corresponding phase.

### 2.4. Statistical Analyses

The frequency at which piglets died or were culled from the population over the period of nursery, that is, the incidence rate per 1000 piglets-day at risk (***I***), was estimated as [14]:

$$I=\frac{\mathrm{number}\;\mathrm{of}\;\mathrm{dead}/\mathrm{culled}\;\mathrm{piglets}\;\mathrm{occurring}\;\mathrm{in}\;a\;\mathrm{period}\;\mathrm{of}\;\mathrm{time}\;\times 1000}{\mathrm{sum}\;\mathrm{of}\;\mathrm{times}\;\left(\mathrm{in}\;\mathrm{days}\right)\;\mathrm{at}\;\mathrm{risk}\;\mathrm{of}\;\mathrm{dying}/\mathrm{being}\;\mathrm{culled}\;\mathrm{of}\;\mathrm{all}\;\mathrm{piglets}}$$

*I* was calculated for each group and for each phase of the nursery period (pre-starter and starter). The relative risk (RR), i.e., the ratio of the *I* in the NVG (INVG) to the *I* in the VG (IVG), and their corresponding 95% confidence intervals (95%CI) were further estimated for the pre-starter and the starter phases.

The pen was used as the experimental unit for comparison of growth performance data (ADG, ADFI) between groups. For this purpose, analysis of variance using the GLM procedure of SAS was used, and group differences were tested using Tukey’s test [15]. Statistical significance was set at *p* < 0.05 for all analyses.

## 3. Results

A total of 30 piglets out of the 1344 included in the study died or were culled during the nursery period. For 25 of them, death occurred during the starter period (43–63 days of age). A description of the number of piglets dead or culled and the clinical signs observed are presented in Table 2. Overall, diarrhea (*n* = 10) was the most frequent cause of piglet death/culling, followed by wasting (*n* = 9).

The *I* of death/culled piglets, although low, was significantly higher for the NVG compared with the VG (1.1 vs. 0.21 1000 piglets-day at risk; *p* < 0.001) during the nursery period. Thus, the RR of dying/being culled for a pig in the NVG was around 5 times higher than that of the VG group (5.04; 95%CI: 3.89–6.54). This risk was negligible for the pre-starter period (28–42 days of life) but increased to almost 12 times when only the starter period (43–63 days) was considered (Table 3). This difference between the pre-starter and the starter periods remained significant even when comparing deaths and runts separately (data not shown).

Body weight at the beginning of the study was not significantly different between the VG and the NVG group (7.65 vs. 7.54 kg, respectively; *p* = 0.26). ADG and ADFI for the pre-starter and starter periods are presented for the VG and NVG in Table 4. The ADG and ADFI of the starter period were significantly higher in the VG group compared with those of the NVG. 

## 4. Discussion

Edema disease caused by Stx2e-producing strains of *E. coli* can be a significant economic disorder in a pig production farm [12]. The cost of associated outbreaks depends on the mortality rate and weight of the pigs that die (the older the pigs, the more expensive the consequences). In addition to these direct losses, a possible immunosuppressive effect of the Stx2e toxin is also suspected [16], which may affect whether piglets thrive properly. 

Vaccination is considered one of the main alternatives for protecting the piglets against this disease, avoiding the use of antimicrobials that may trigger the selection for antimicrobial resistance [17]. In this study, a vaccine against Stx2e was used in a farm where STEC was previously confirmed, and vaccinated and non-vaccinated pigs were monitored along the whole nursery period. A significantly lower (5 times less) number of losses (dead or culled pigs) were observed among the vaccinated piglets compared with those in non-vaccinated group during the nursery period. 

Some of the losses within the NVG were associated with clinical signs that could be related to ED, such as neurological disorders, sudden death, wasting (runts), and diarrhea [5], but no laboratory analyses were specifically performed to confirm the etiologic agent of the clinical signs. Thus, it is likely that some of them may be attributed to other infectious agents potentially present in the farm (i.e., *Glaesserella parasuis*, *Streptococcus suis*, or *Pasteurella multocida*). It cannot be ruled out that part of the positive vaccination results might be attributed to NSE or trained immunity, since specific immunity is initiated by non-specific innate immune mechanisms [10,11]. In addition, aluminum hydroxide-based adjuvants, the type of adjuvant in the tested vaccine, may also contributed to the stimulation of these innate immune responses [18]. 

One of the most common clinical scenarios of ED is associated with losses occurring from 2 to 4 weeks postweaning, after the change of feed from pre-starter to starter [19]. Other factors that have been associated with increase of ED incidence are the withdrawal of ZnO or of antibiotics from feed, along with any stressor agent, such as transportation, mixing of pigs, presence of other infections, etc. [5]. In the present study, both the NVG and the VG experienced a change in feed (pre-starter to starter) and the withdrawal of the in-feed administration of ZnO at the same time, which was routinely used for two weeks after weaning to limit proliferation of enteropathogenic *E. coli* [20]. However, this pattern of losses was only observed in the NVG, not in the VG. The incidence rate of mortality/culling was significantly higher during the starter period for the NVG, which suffered from almost 12 times higher risk of losses (RR = 11.7; 95%CI: 2.7–49.5) compared with the VG. Thus, it appears that vaccination against Stx2e may have prevented further losses in the VG during the starter phase of the nursery period.

Although a direct comparison between mortality (deaths plus runts) in this VG and mortality in a previous period, in which a combination of colistin and ZnO was used, could not be properly conducted because the groups may differ, it is interesting to highlight that mortality was virtually the same during the starter phase for both groups (0.14 vs. 0.15 1000 piglets-day at risk, respectively). The vaccine used in the present study, based on a genetically modified recombinant Stx2e, has already been shown to be apparently effective in reducing the use of colistin and the mortality rate (MR) during the nursery period [12,21]. Thus, this study supports previous results suggesting that vaccination against Stx2e may reduce mortality and may likely be used as an alternative to the use of colistin.

Effects of vaccination were noted on performance parameters as well. During the starter period, the ADG in the VG was significantly better than that in the NVG (387.3 g/d vs. 375 g/d, *p* = 0.04). Better ADG results for the vaccinated groups were also found in the nursery period in the abovementioned German study of Lillie-Jaschniski et al. (2013). These results are of great importance, as ADG during the nursery period has a major impact on subsequent growth performance [22]. 

Interestingly, ED occurs mostly in the best pigs, those that also show higher feed intake, which would favor the imbalance of microbiota and the proliferation of pathogenic *E. coli* [23,24]. In this study, during the starter period, the ADFI for the VG was significantly larger than that in the NVG (563.6 g/d vs. 541.4 g/d, *p* = 0.010). It appears that despite this higher feed consumption in the VG, along with other risk factors present such as the withdrawal of ZnO and the feed change, the vaccine would have helped to keep mortality significantly lower compared with the NVG.

## 5. Conclusions

Considering that the metaphylactic use of antibiotics is not advisable and that ZnO will be banned by 2022, alternatives for controlling the effects of ED during the nursery period should be explored. The present study showed that after vaccination against Stx2e in a farm with previous history of ED, postweaning losses were reduced during the whole nursery period, and performance parameters improved in the starter period, thus suggesting that this approach could be a useful alternative to colistin and/or ZnO.

## Figures and Tables

**Table 1:** Performance parameters and incidence rate (*I*; 1000 piglets-day at risk) of animal losses during the nursery period in the farm for the period before the beginning of the study, when both ZnO and colistin were routinely used.

Average Daily Gain (g/d)		Average Daily Feed Intake (g/d)		*I*	
Pre-starter	Starter	Pre-starter	Starter	Pre-starter	Starter
171.7	390.7	231.4	554.6	0.149	0.149

Pre-starter: 28–42 days; Starter: 43–63 days.

**Table 2:** Number of piglet losses and major clinical signs associated with them during the nursery in each study group.

Clinical Signs/Conditions	Pre-Starter		Starter		
	VG	NVG	VG	NVG	Total (Percentage)
Diarrhea	1	2	1	6	10 (33.3%)
Runts	1		1	7	9 (30.0%)
Respiratory problems				5	5 (16.7%)
Nervous signs				2	2 (6.7%)
Lameness				1	1 (3.3%)
Sudden death				1	1 (3.3%)
Accident	1				1 (3.3%)
Unknown				1	1 (3.3%)
Subtotals	3	2	2	23	
Total	5		25		30 (100%)

Pre-starter: 28–42 days; Starter: 43–63 days. VG (vaccinated group against ED); NVG (non-vaccinated group).

**Table 3:** Estimates of incidence rate (*I*; 1000 piglets-day at risk) for the non-vaccinated (NVG) and vaccinated (VG) groups for each period of nursery (pre-starter and starter) and corresponding relative risk (RR) between them.

Group	Pre-Starter (28–42 Days of Life)						Starter (43–63 Days of Life)					
	**D**	**R**	**TL**	***I***	**RR (95%CI)**	***p***	**D**	**R**	**TL**	***I***	**RR (95%CI)**	***p***
NVG	2	0	2	0.21	0.66 (0.4–1.2)	0.84	16	7	23	1.6	11.7 (2.7–49.5)	<0.001
VG	2	1	3	0.32			1	1	2	0.14		

VG (vaccinated group against ED); NVG (non-vaccinated group). *p* (*p*-value) < 0.05 is indicative of significant differences for a given period. D (No. deaths); R (No. runts); TL (No. of total losses).

**Table 4:** Average daily weight gain and average daily feed intake results for the different studied groups.

Study Groups	Average Daily Gain (g/d)		Average Daily Feed Intake (g/d)	
	**Pre-Starter** **28–42 Days**	**Starter** **43–63 Days**	**Pre-Starter** **28–42 Days**	**Starter** **43–63 Days**
VG	171.4	387.3	227.3	563.6
NVG	176.6	375.0	230.5	541.4
*p*	0.287	0.040	0.472	0.010

VG (vaccinated group against ED); NVG (non-vaccinated group). *p* (*p*-value) < 0.05 is indicative of significant differences for a given period.

## Data Availability

Data available upon request.
